# Improved quantification of abnormal aortic flow in 3D compared to standard 2D approach

**DOI:** 10.1186/1532-429X-15-S1-P232

**Published:** 2013-01-30

**Authors:** Monica Sigovan, Michael D Hope, Jarrett Wrenn, Petter Dyverfeldt, David Saloner

**Affiliations:** 1 Radiology and Biomedical Imaging, UCSF, San Francsico, CA, USA; 2 Linköping University, Linköping, Sweden

## Background

With 4D MR flow imaging, eccentric flow has been reported in patients with bicuspid aortic valve (BAV) and investigated as a risk factor for ascending aortic (AsAo) aneurysms [1]. We have demonstrated that a new parameter, normalized displacement, quantifies the degree of flow eccentricity in the AsAo [2]. However, it only offers information on a single location in the AsAo. The aim of the current work was to extend the normalized displacement parameter to utilize the entire 4D flow information. We developed a 3D analysis method and analyzed the degree and pattern of eccentric systolic flow in the AsAo of individuals with different aortic pathologies.

## Methods

A 3D Phase-Contrast MRI pulse sequence was used to assess blood flow in the thoracic aorta. MR imaging was performed at 1.5T (Siemens Avanto, and Signa, GE). Informed consent was obtained from all participants.

**Figure 1:**
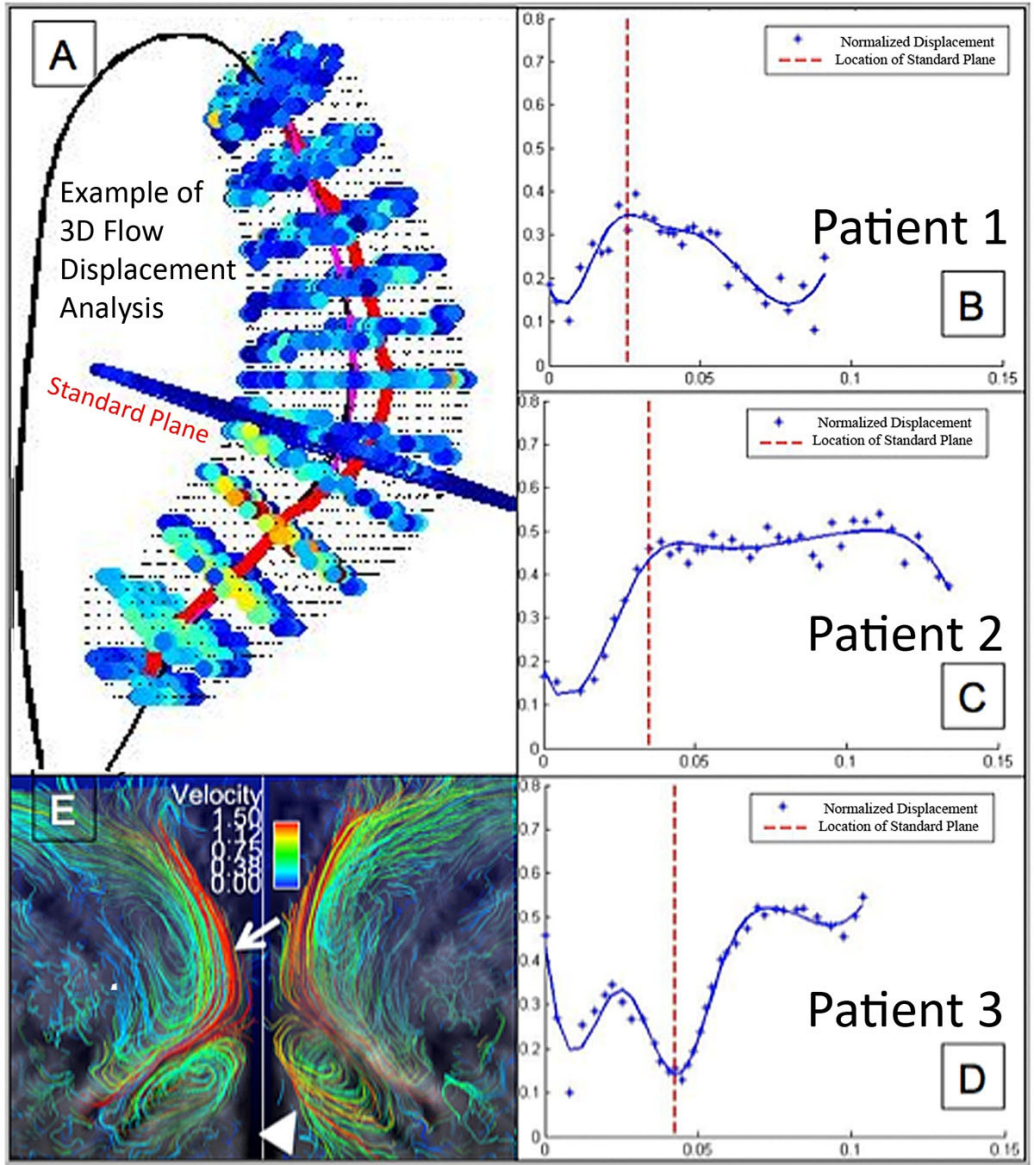
Example of the 3D flow displacement analysis results: A - cross-sections containing speed information are automatically placed along the vessel centerline (black), and normalized displacement is calculated for each cross-section (red line); Plots B to D present the variation of the normalized displacement for different patients. A standard plane was placed based on anatomic landmarks (red dashed lines). In some cases the standard plane can entirely miss the abnormal flow (D). Streamline visualization of the systolic flow pattern presented in inset E explains the displacement plot in D. recirculation in the aortic root (arrow head) results in an early displacement peak, but then pushes flow centrally at the level of the standard plane, only to become markedly eccentric at the level of the pulmonary artery (arrow).

Manual segmentation of the aorta was performed, and the vessel centerline was subsequently obtained. The automated flow analysis of the AsAo segment was performed in Matlab (The MathWorks Inc, Natick, MA). Cross-sectional planes were automatically placed along the centerline at equal spacing (Figure 1A). For each cross-section, normalized displacement, defined as the distance between the center of the lumen and the center of velocity of the forward flow, normalized to the diameter of the cross-section, was obtained. Finally, the Area under the curve of the Normalized Displacement plotted against the distance along the centerline was calculated, and normalized to the length of the investigated segment.

## Results

28 subjects were divided in 5 groups based on their aortic pathology: Normal (n=6), BAV without aortic stenosis (AS) or dilatation (n=7), TAV with AS, no dilatation (n=3), TAV with dilatation, no AS (n=6), and TAV with both AS and dilatation (n=6).

Representative plots of normalized displacement from multiple cross-sections as a function of distance along the AsAo are presented in Figure 1 for a BAV subject (B), and two TAV + AS & Dilation subjects (C, D). Flow patterns had similar characteristics within a group of subjects. Generally, presence of AS was related to a rapid increase in normalized displacement, followed by a plateau. Normalized displacement variation along the AsAo for BAV subjects presented an increase followed by an immediate decrease of values, with absence of a plateau.

**Figure 2:**
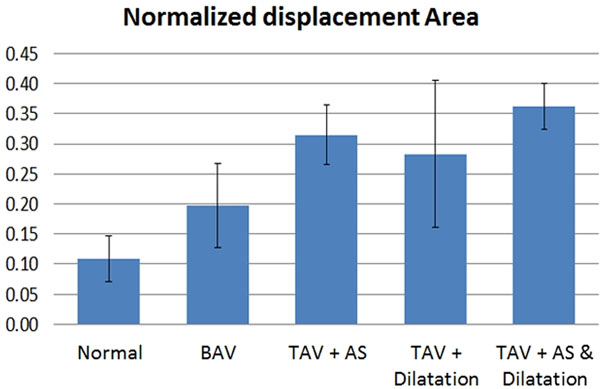
Average values of the area of normalized displacement for each of the 5 groups. Subjects with normal flow present the lowest values of displacement.

Average values of the area under the curve of normalized displacement were: 0.11±0.04 (Normal), 0.2±0.07 (BAV), 0.31±0.05 (TAV + AS), 0.28±0.12 (TAV + Dilatation), and 0.36±0.04 (TAV + AS & Dilatation) (Figure 2).

## Conclusions

The improved 3D flow displacement analysis allows visualization of the flow pattern along the ascending aorta, and enables detection of characteristics specific to different aortic pathologies. Furthermore, the 3D method better captures abnormal flow with aortic valve disease than the standard 2D analysis.

## Funding

Covidien/Radiologic Society of North America Research Scholar Grant 2012-2014.
